# MicroRNA co-expression networks exhibit increased complexity in pancreatic ductal compared to Vater's papilla adenocarcinoma

**DOI:** 10.18632/oncotarget.22184

**Published:** 2017-10-31

**Authors:** Tommaso Mazza, Massimiliano Copetti, Daniele Capocefalo, Caterina Fusilli, Tommaso Biagini, Massimo Carella, Antonio De Bonis, Nicola Mastrodonato, Ada Piepoli, Valerio Pazienza, Evaristo Maiello, Fabio Francesco di Mola, Pierluigi di Sebastiano, Angelo Andriulli, Francesca Tavano

**Affiliations:** ^1^ Unit of Bioinformatics, Research Hospital, San Giovanni Rotondo 71013, Italy; ^2^ Unit of Biostatistics, Research Hospital, San Giovanni Rotondo 71013, Italy; ^3^ Medical Genetics Unit, Research Hospital, San Giovanni Rotondo 71013, Italy; ^4^ Department of Surgery, Research Hospital, San Giovanni Rotondo 71013, Italy; ^5^ Division of Gastroenterology and Research Laboratory, San Giovanni Rotondo 71013, Italy; ^6^ Department of Oncology IRCCS “Casa Sollievo della Sofferenza”, Research Hospital, San Giovanni Rotondo 71013, Italy; ^7^ Division of Surgical Oncology “SS Annunziata” Hospital, Chieti 66100, Italy; ^8^ Department of Cellular Biotechnologies and Haematology, Sapienza University of Rome, Rome 00161, Italy

**Keywords:** microRNA, pancrearic ductal adenocarcinoma, ampullary carcinoma

## Abstract

MiRNA expression abnormalities in adenocarcinoma arising from pancreatic ductal system (PDAC) and Vater's papilla (PVAC) could be associated with distinctive pathologic features and clinical cancer behaviours. Our previous miRNA expression profiling data on PDAC (n=9) and PVAC (n=4) were revaluated to define differences/similarities in miRNA expression patterns. Afterwards, in order to uncover target genes and core signalling pathways regulated by specific miRNAs in these two tumour entities, miRNA interaction networks were wired for each tumour entity, and experimentally validated target genes underwent pathways enrichment analysis.

One hundred and one miRNAs were altered, mainly over-expressed, in PDAC samples. Twenty-six miRNAs were deregulated in PVAC samples, where more miRNAs were down-expressed in tumours compared to normal tissues. Four miRNAs were significantly altered in both subgroups of patients, while 27 miRNAs were differentially expressed between PDAC and PVAC.

Although miRNA interaction networks were more complex and dense in PDAC than in PVAC, pathways enrichment analysis uncovered a functional overlapping between PDAC and PVAC. However, shared signalling events were influenced by different miRNA and/or genes in the two tumour entities.

Overall, specific miRNA expression patterns were involved in the regulation of a limited core signalling pathways in the biology landscape of PDAC and PVAC.

## INTRODUCTION

Pancreatic cancer is one of the most lethal malignancies which ranks fourth among the most prominent causes of cancer-related deaths in Western countries [1]. Although the exocrine pancreas consists of a very small ductal system, 90% of pancreatic neoplasms are ductal in origin and are known as pancreatic ductal adenocarcinomas (PDAC) [2]. The propensity of PDAC to invade nearby organs and surrounding tissues, as well as the lack of effective means for screening and early detection, contribute to a significant delay in diagnosis, which thus usually occurs only after having reached advanced disease stages [3]. Consequently, the percentage of resectable patients is low and the average survival of these patients is approximately 12 to 20 months, with a high probability of relapse [2–4].

Adenocarcinoma of ampulla of Vater (PVAC) differs from PDAC in prognosis and in how it occurs. Based on its localization, PVAC is usually detected relatively early: the confluence of the common bile duct and the main pancreatic duct at the ampulla are signs that the tumours that arise at this location have the potential to obstruct two major organs and to result in relatively early onset of symptoms, which, most commonly, are biliary obstruction and pancreatitis. This explains why these lesions account for about half of all resectable pancreatic neoplasms, often before disease spreading to lymph nodes, and why PVAC patients have a consistently better survival than patients with PDAC [5–7]. However, the difference in survival is not entirely explained by the lower frequency of lymph node involvement in PVAC. In fact, the survival for node-positive patients is still better than in PDAC and this difference may be explained by biological, particularly molecular, differences between the two cancer entities.

Recently, microRNAs (miRNAs) have grabbed a wide attention for their pivotal involvement in cancer development [8]. MiRNAs influence various biological processes, including cell proliferation, cell death and stress resistance, mainly by of gene expression [9]. The relationship between the miRNA expression dysregulation and the survival of patients affected by pancreatic adenocarcinoma was already reported in PDAC [10–13]. Conversely, little is known about the potential involvement of miRNAs in the onset and development of PVAC.

Our hypothesis is that several molecular characteristics of PDAC and PVAC, including the miRNA expression abnormalities, could be associated with distinctive pathologic features and clinical behaviours. Overall the aim of this study was to make a comparison between these two tumour entities on the pathogenetic level. For this purpose we defined differences and similarities of miRNA expression patterns of patients with PDAC and PVAC, up to respective target genes and signalling pathways. To this extent, we attempted to unravel the landscape of miRNA expression among PDAC and PVAC using co-expression networks as a proxy for highlighting the main differences between them and their healthy counterpart, as correlation networks are often used as discovery tools [14].

## RESULTS

### MiRNAs differentially expressed in tumour *versus* normal tissues from PDAC and PVAC patients

One hundred and one miRNAs, out of 1105 miRNAs assayed in our array experiments, were differentially expressed in PDAC tumours, compared to their adjacent normal tissue samples (68 over-expressed and 33 down-regulated). MiR-887 was the most significantly altered miRNA (p=9.21×10^−5^), (Table 1).

**Table 1:** MicroRNA differentially expressed, by *t*-test, in paired normal and tumour tissues from patients with pancreatic ductal adenocarcinoma (PDAC)

miRNA	Fold-change (T vs N)	P-value	miRNA	Fold-change (T vs N)	P-value
hsa-miR-887	1.43	9.21E-05	hsa-miR-199b-5p	1.57	0.0263
hsa-miR-125a-5p	12.94	0.0021	hsa-let-7d^*^	−1.70	0.0268
hsa-miR-1321	−1.50	0.0024	hsa-miR-503	1.80	0.0272
hsa-miR-744	2.25	0.0037	hsa-miR-1244	2.46	0.0274
hsa-miR-500^*^	3.30	0.0039	hsa-miR-1254	−1.59	0.0279
hsa-miR-526a	1.34	0.0040	hsa-miR-221	3.62	0.0283
hsa-miR-214	4.24	0.0040	hsa-miR-27b^*^	2.02	0.0285
hsa-miR-1267	−2.26	0.0042	hsa-miR-708	3.33	0.0292
hsa-miR-181a	5.81	0.0043	hsa-miR-132	2.80	0.0298
hsa-miR-1249	−1.86	0.0048	hsa-let-7i	3.16	0.0306
hsa-miR-125a-3p	1.96	0.0048	hsa-miR-92b	1.74	0.0306
hsa-let-7e	12.59	0.0051	hsa-miR-24	6.33	0.0310
hsa-miR-1237	−1.68	0.0074	hsa-miR-199a-5p	4.90	0.0315
hsa-miR-181a^*^	−2.60	0.0081	hsa-miR-29b	1.36	0.0318
hsa-miR-134	1.84	0.0083	hsa-miR-520a-3p	−1.40	0.0331
hsa-miR-29b-1^*^	1.52	0.0083	hsa-miR-378^*^	1.66	0.0340
hsa-miR-132^*^	−2.47	0.0084	hsa-miR-143	5.07	0.0340
hsa-miR-23a	7.12	0.0087	hsa-miR-16-2^*^	−1.46	0.0340
hsa-miR-559	−2.59	0.0095	hsa-miR-181c^*^	−2.26	0.0341
hsa-miR-339-5p	2.52	0.0098	hsa-miR-30b	−1.92	0.0346
hsa-miR-154^*^	−1.87	0.0101	hsa-miR-379	1.96	0.0352
hsa-miR-140-5p	1.65	0.0101	hsa-miR-136^*^	−1.43	0.0353
hsa-miR-181b	4.82	0.0105	hsa-miR-183^*^	−1.20	0.0373
hsa-miR-331-3p	2.56	0.0114	hsa-miR-100	2.43	0.0380
hsa-miR-92a	4.00	0.0124	hsa-miR-145	5.37	0.0380
hsa-let-7a	8.04	0.0141	hsa-miR-550	1.40	0.0392
hsa-miR-517^*^	−1.40	0.0153	hsa-miR-26a	5.03	0.0395
hsa-miR-30c	−1.81	0.0155	hsa-miR-423-3p	1.85	0.0405
hsa-miR-1301	2.14	0.0156	hsa-miR-373	−1.44	0.0412
hsa-miR-939	1.82	0.0159	hsa-miR-107	5.30	0.0416
hsa-miR-1227	−2.01	0.0164	hsa-miR-219-1-3p	−1.66	0.0422
hsa-miR-487b	−2.12	0.0170	hsa-miR-182^*^	1.24	0.0423
hsa-miR-502-3p	3.40	0.0171	hsa-miR-103	2.71	0.0425
hsa-miR-125b	4.06	0.0175	hsa-miR-558	−2.60	0.0427
hsa-miR-99b	2.86	0.0176	hsa-miR-23b	6.00	0.0438
hsa-miR-32	−1.20	0.0176	hsa-miR-99a	2.80	0.0440
hsa-miR-199b-3p	4.99	0.0178	hsa-let-7g	1.75	0.0444
hsa-miR-374a	−2.39	0.0184	hsa-miR-361-5p	3.12	0.0445
hsa-miR-21	4.33	0.0185	hsa-miR-1246	2.28	0.0456
hsa-let-7d	4.73	0.0191	hsa-miR-625^*^	−1.61	0.0457
hsa-miR-1304	−1.28	0.0196	hsa-miR-130a^*^	−1.53	0.0457
hsa-miR-199a-3p	5.55	0.0198	hsa-miR-146a	2.67	0.0459
hsa-miR-498	1.51	0.0207	hsa-miR-663b	1.71	0.0459
hsa-miR-324-3p	1.72	0.0226	hsa-miR-10a	1.96	0.0464
hsa-miR-99b^*^	1.71	0.0229	hsa-miR-431	−1.42	0.0465
hsa-miR-181d	2.32	0.0238	hsa-miR-27b	2.62	0.0469
hsa-miR-411	−2.18	0.0242	hsa-miR-1259	−1.42	0.0476
hsa-miR-34a	2.81	0.0242	hsa-miR-222	3.74	0.0485
hsa-miR-195	3.24	0.0250	hsa-miR-30d	−1.34	0.0487
hsa-miR-196a	1.34	0.0252	hsa-miR-337-3p	−1.39	0.0494
hsa-miR-382	2.37	0.0262			

In PVAC, 26 miRNAs were differentially expressed in tumours compared to normal tissues (Table 2). In details, 7 miRNAs showed higher expression levels in tumour than normal tissues, whereas 19 miRNAs were down-regulated. In this cohort, miR-323-3p showed the most significantly altered expression in tumour samples (p=0.0042), (Table 2).

**Table 2:** MicroRNA differentially expressed, by *t*-test, in paired normal and tumour tissues from patients with adenocarcinoma of papilla of Vater (PVAC)

miRNA	Fold-change (T vs N)	P-value
hsa-miR-323-3p	1.59	0.0042
hsa-miR-525-5p	−1.22	0.0111
hsa-miR-199b-5p	−1.25	0.0120
hsa-miR-30d^*^	−1.27	0.0130
hsa-miR-339-3p	−3.35	0.0202
hsa-miR-490-5p	−1.34	0.0208
hsa-miR-1305	−1.69	0.0232
hsa-miR-1270	−1.35	0.0273
hsa-miR-1254	−2.22	0.0292
hsa-miR-643	1.55	0.0303
hsa-miR-29b-2^*^	1.73	0.0309
hsa-miR-509-5p	−1.10	0.0316
hsa-miR-200c^*^	1.38	0.0335
hsa-miR-410	−1.51	0.0337
hsa-miR-563	−1.30	0.0343
hsa-miR-187^*^	−1.49	0.0345
hsa-miR-889	−6.53	0.0352
hsa-miR-551a	−1.22	0.0394
hsa-miR-30b^*^	−1.58	0.0397
hsa-miR-140-5p	1.43	0.0399
hsa-miR-1280	−3.97	0.0401
hsa-miR-548b-3p	1.29	0.0423
hsa-miR-1228^*^	−4.07	0.0432
hsa-miR-551b^*^	−1.17	0.0442
hsa-miR-548l	−1.37	0.0479
hsa-miR-103	15.43	0.0485

Table 3 reports differentially expressed miRNAs between tumour and normal tissues for both PDAC and PVAC. MiR-140-5p, miR-103, miR-1254, miR-199b-5p were significantly deregulated in both the subgroups of patients.

**Table 3:** MicroRNA showing significant or a trend of alteration in tumour compared to normal tissues samples in both pancreatic ductal adenocarcinoma (PDAC) and adenocarcinoma of papilla of Vater (PVAC)

	PDAC		PVAC	
	Fold-change (T vs N)	P-value	Fold-change (Tvs N)	P-value
hsa-miR-140-5p	1.65	**0.0101**	1.43	**0.0399**
hsa-miR-103	2.71	**0.0425**	15.43	**0.0485**
hsa-miR-1254	−1.59	**0.0279**	−2.22	**0.0292**
hsa-miR-199b-5p	1.57	**0.0263**	−1.25	**0.0120**
hsa-miR-1244	2.46	**0.0274**	7.48	*0.0579*
hsa-miR-146a	2.67	**0.0459**	5.02	*0.0593*
hsa-miR-30d	−1.34	**0.0487**	−4.56	*0.0556*
hsa-miR-1259	−1.42	**0.0476**	1.25	*0.0606*
hsa-miR-1304	−1.28	**0.0196**	1.15	*0.0828*
hsa-miR-525-5p	−1.18	*0.0837*	−1.22	**0.0111**
hsa-miR-643	−1.13	*0.0903*	1.55	**0.0303**
hsa-miR-1207-5p	2.08	*0.0722*	2.08	*0.0771*
hsa-miR-138-1^*^	1.54	*0.0807*	2.10	*0.0526*
hsa-let-7f-1^*^	−1.58	*0.0589*	−1.51	*0.0623*
hsa-miR-629^*^	−2.25	*0.0716*	−5.11	*0.0722*
hsa-miR-1260	−1.63	*0.0809*	−2.02	*0.0982*
hsa-miR-302a^*^	−1.64	*0.0808*	1.24	*0.0546*

To ascertain the eventual existence of orthologous miRNAs, and to use them as internal methodological control, the human miRNAs that were found altered in PDAC and PVAC samples were checked in 71 other organisms probed in the microarray assay. All deregulated orthologous of human miRNAs showed a significant differential expression with similar fold-changes in these organisms, with the only exception of miR-182^*^ and miR-323-3p in the PDAC and PVAC subgroups of patients, respectively (Supplementary Table 1A-B).

### MiRNAs differentially expressed in PDAC *versus* PVAC

In order to identify differentially expressed miRNAs between PDAC and PVAC patients, expression levels in paired tumour and normal tissues from PDAC and PVAC were compared. Twenty-seven miRNAs showed a significantly altered expression between PDAC and PVAC (Table 4). In details, 19 and 8 miRNAs were over- and down-regulated in PDAC compared to PVAC, respectively, with miR-889 and miR-323-3p showing the most relevant alteration (over- and down- expression, respectively) in PDAC *vs* PVAC.

**Table 4:** MicroRNA differentially expressed in paired normal and tumour tissues, by *t*-test, in pancreatic ductal adenocarcinoma (PDAC) compared to adenocarcinoma of papilla of Vater (PVAC)

miRNA	Fold change (PDAC vs PVAC)	P-value
hsa-miR-323-3p	−2.13	0.0006
hsa-miR-199b-5p	2.04	0.0024
hsa-miR-1259	−1.93	0.0024
hsa-miR-1304	−1.50	0.0067
hsa-miR-550	2.30	0.0130
hsa-miR-122	−1.55	0.0145
hsa-miR-302a^*^	−2.08	0.0157
hsa-miR-922	−1.99	0.0183
hsa-miR-125a-3p	2.33	0.0195
hsa-miR-643	−1.83	0.0236
hsa-miR-1301	2.50	0.0249
hsa-miR-518c^*^	1.41	0.0259
hsa-miR-889	7.42	0.0317
hsa-miR-516a-3p	1.66	0.0332
hsa-miR-939	1.69	0.0335
hsa-miR-125b-1^*^	1.99	0.0336
hsa-miR-187^*^	1.73	0.0339
hsa-miR-18b^*^	3.09	0.0349
hsa-miR-548b-3p	−2.07	0.0369
hsa-miR-1270	1.54	0.0369
hsa-miR-593	1.31	0.0382
hsa-miR-410	1.64	0.0415
hsa-miR-551b^*^	1.58	0.0435
hsa-miR-23b^*^	1.60	0.0452
hsa-miR-490-5p	1.86	0.0456
hsa-miR-339-3p	2.78	0.0457
hsa-miR-450a	1.58	0.0496

Human miRNAs that were altered both in PDAC and PVAC were checked for the existence of orthologs in 71 organisms: all deregulated orthologs of human miRNAs showed a significant differential expression with close fold-changes, with the only exception of miR-323-3p (Supplementary Table 1C).

### Networks of correlated miRNAs in normal and tumour tissue samples

#### PDAC

**Figure 1:**
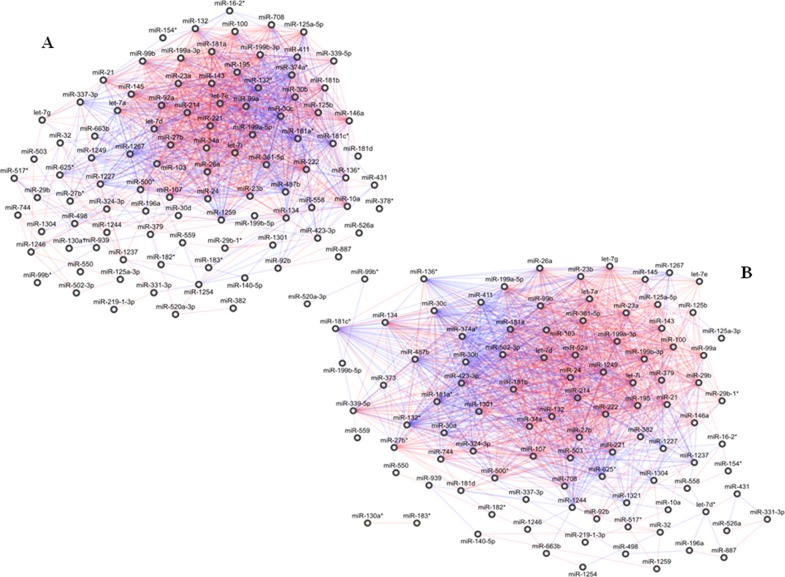
Correlation network between microRNA altered in tissue samples from patients with pancreatic ductal adenocarcinoma (PDAC) **(A)** Normal tissues; global geometric attributes: connected components 1, network diameter 6, network centralization 0.27, shortest paths 9506 (100%), characteristic path length 2.35, average number of neighbours 25.14, network density 0.26, and network heterogeneity 0.77. **(B)** Tumour tissues; global geometric attributes: connected components 3, network diameter 6, network centralization 0.32, shortest paths 89.38 (90%), characteristic path length 2.18, average number of neighbours 28.68, network density 0.29, and network heterogeneity 0.72. Direct and inverse correlations are in red and blue, respectively.

Networks of normal and tumour tissues were quite complex. The *normal* network was made of 98 miRNAs, wired by 1.232 correlation edges (Figure 1A). The *diseased* network was composed by 100 miRNAs connected by 1.434 links (Figure 1B). One thousand and one hundred and ninety and 542 out of the 1434 and 1232 links were preserved in the public dataset E-GEOD-60978. In details, 812 and 439 of these links had concordant correlation signs in PDAC and normal tissues, respectively, compared to our results (Supplementary Information #Sheet A).

In PDAC, normal and tumour networks preserved several unchanged correlations for most of their miRNAs: only 4 were not shared by the two networks, including 1 (miR-348^*^) and 3 (miR-let-7d^*^, miR-1321, miR-373) out of the 98 and the 100 connected miRNAs in the normal and in the tumour networks, respectively. Conversely, 97 miRNAs were shared by the two networks including 65 linked by 775 edges, which represented more than 50-60% of the total number of the edges of both the networks, and 32 miRNAs which did not share any link between the normal and the tumour networks (Supplementary Table 2A).

Two (miR-1321 and miR-373-3p) out of the 3 miRNAs specifically belonging to the PDAC tumour network participate to the *ABC transporters* signalling pathway (Supplementary Table 3). Among the miRNAs shared between normal and tumour networks (Supplementary Table 2B), those that maintained the same links in the two networks (n=65) enriched 80 cancer-related pathways, while 35 cancer-associated signalling events emerged for 7 out of the 32 miRNAs which did not share any links between normal and tumour networks. These miRNAs (miR-331-3p, miR-1246, miR-382, miR-558, miR-181d, miR-1301, miR-559) exhibited high fold change values, and closely enriched 3 out of the 35 above mentioned signalling pathways.

As listed in Supplementary Table 2C, the most relevant signalling pathways for each subgroup of miRNAs were identified. In details, the pathways of the *FOXO family* and of *MAPK* were the most significantly impacted by the 65 shared miRNAs. The sets of 7 and 65 aforesaid miRNAs equally enriched the *regulation of nuclear SMAD2/3* pathway, while ERBB1 and c-MET signalling pathways were enriched mainy by the 7 differentially connected miRNAs. The major pathways enriched by the 7 differentially connected miRNAs were the ERBB1 and c-MET signalling pathways. In addition, the *Notch mediated HES/HEY* network resulted to be enriched solely by the subgroup of the 7 miRNAs.

#### PVAC

**Figure 2:**
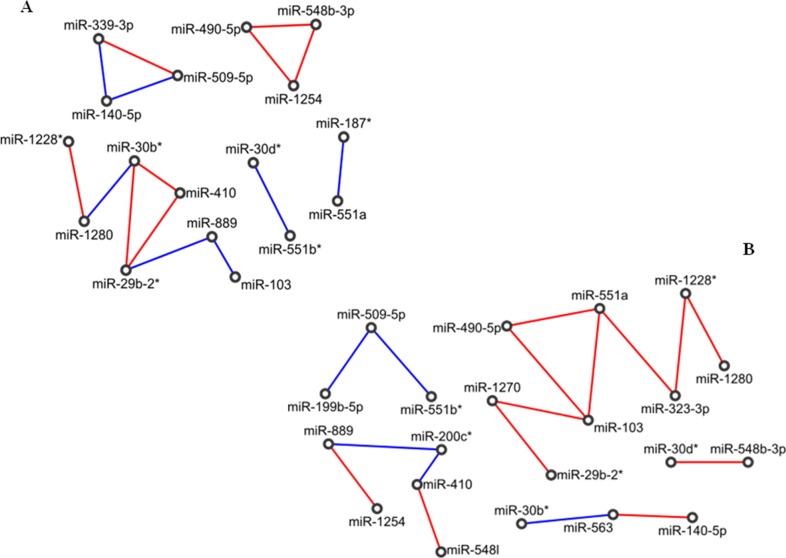
Correlation network between microRNA altered in tissue samples from patients with adenocarcinoma of papilla of Vater (PVAC) **(A)** Normal tissues; **(B)** tumour tissues. Direct and inverse correlations are in red and blue, respectively.

Both normal-adjacent and tumour PVAC networks were much smaller than the corresponding PDAC networks. In particular, the normal network was made of 17 nodes and 15 edges. Topologically, this network was characterized by a long chain of expression correlations among 7 miRNAs (miR-103, miR-889, miR-29b-2^*^, miR-410, miR-30b^*^, miR-1280 and miR-1228^*^), by 2 closed groups (triangles) including miR-548b-3p, miR-1254, miR-490-5p (triangle 1) and miR-339-3p, miR-509-5p, miR-140-5p (triangle 2), respectively, and by two pairs of miRNAs (miR-551b^*^/miR-30d^*^ and miR-551a/miR-187^*^), (Figure 2A).

The tumour network was made of 21 miRNAs linked by 17 edges. No closed groups resulted, whereas it included a long chain of 8 miRNAs (miR-1280, miR-1228^*^, miR-323-3p, miR-551a, miR-490-5p, miR-103, miR-1270 and miR-29b-2^*^), three shorter chains of 5 miRNAs (miR-1254, miR-889, miR200c^*^, miR-410 and miR-548l) and 3 miRNAs (miR-563/miR-30b^*^/miR-140-5p and miR-551b^*^/miR-509-5p/miR-199b-5p), and one pair of miRNAs (miR-548b-3p/miR-30d^*^). In particular, the longest chain was made of an odd number (five) of consecutive inverse correlations (miR-1270, miR-103, miR-490-5p, miR-551a, miR-323-3p, miR-1228^*^), of which miR-103 was the only miRNA to be highly expressed (Figure 2B).

Comparing normal with tumour networks of PVAC samples, we found 15 miRNAs and only 1 edge in common, whereas 6 miRNAs (miR-1270, miR-323-3p, miR-200c^*^, miR-548I, miR-563, miR-199b-5p) were included only in the diseased network. By pathway enrichment analysis, 3 out of these 6 miRNAs were involved in the *LICAM interactions* process [miR-199b-5p (AKN2, CLTC, LAMC1), miR-323a-3p (ANK2, DCX, KCNQ3, KIAA1598, LAMC1, NRP2, SCN2A, SPTBN1), miR-563 (ITGA9, SPTBN2)], and in the *axon guidance* signalling pathway [miR-199b-5p (ANK2, ARHGEF12, CLTC, GSK3B, LAMC1, MYH9, PLXNA2, RGMA, RGMB, RND1, SEMA6A, SRGAP2), miR-323a-3p (ANK2, CLASP1, CREB1, DCX, ENAH, KCNQ3, KIAA1598, KRAS, LAMC1, MET, NRP2, PAK7, PITPNA, ROCK1, SCN2A, SEMA6D, SPTBN1, SRGAP1), miR-563 (COL1A2, COL3A1, ITGA9, SPTBN2)]. Conversely, miR-1270, miR-200c^*^, miR-548I did not enrich any pathway related to pancreatic cancer.

Focusing on miRNAs with elevate fold-change values in the two networks, namely on miR-1254 (FC=-2.22), miR-1228^*^ (FC=-4), miR-1280 (FC=-3.97), miR-889 (FC=-6.53) and miR-103 (FC=15.4), we noticed that the only common correlation was that between miR-1228^*^ and miR-1280. MiR-889 was directly linked to and inversely correlated with miR-103 in normal tissues, while it was directly correlated with miR-1254 in tumour tissues. However, only miR-103 was directly involved in the regulation of pancreatic cancer-associated processes: *E-cadherin* signalling events (CDH1), *TGF beta* signalling pathway (TGFBR2, APC, CDH1, CREBBP, EP300, SMAD3, TSC2), and altered *TFG-beta SMAD* dependent signalling (TGFBR2, SMAD3, and FBXW7).

Finally, the two triangles of miRNAs were peculiar to the normal network only, and among their validated target genes retrieved by miRTarBase, miRWalk and TarBase 7 there were DICER1 (controlled by miR-548b-3p), TP53 and CDH1 (regulated by miR-140-5p, belonging to the triangle 2). These genes are mostly involved in the perturbation of *DICER1*, *p53*, *p21*, and *E-cadherin* pathways.

### Networks of correlated miRNAs in tumour versus normal tissue samples

#### PDAC

**Figure 3:**
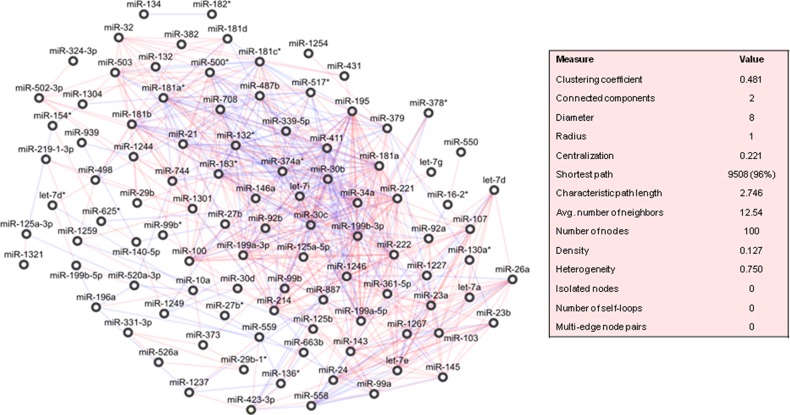
Correlation network between microRNA altered in tumour compared to normal tissue samples from patients with pancreatic ductal adenocarcinoma (PDAC) Direct and inverse correlations are in red and blue, respectively.

A correlation-based network was built with the differentially expressed miRNAs between healthy-adjacent and tumour tissues, and drawn in Figure 3. This was clustered in 5 highly cohesive subgroups of miRNAs: *cluster 1* with 38 miRNAs (density: 0.504, p-value: 1.405E-12); *cluster 2* with 18 miRNAs (density: 0.608, p-value: 2.556E-5); *cluster 3* with 5 miRNAs (density: 0.700, p-value: 0.007); *cluster 4* with 4 miRNAs (density: 0.833, p-value: 0.025), and *cluster 5* with 8 miRNAs (density: 0.571, p-value: 0.077). The complete lists of miRNAs belonging to each cluster is reported in Supplementary Table 4.

A ranking of these miRNAs was assessed by the calculation of an array of topological indices (cf. Methods). We verified that 9 miRNAs (miR-34a, miR-125a-5p, miR-199a-5p, miR-181a, miR-30c, miR-30b, miR-339-5p, miR-214, miR-411) belonging to cluster 1 were top-five ranked for at least one topological index, and that 3 of these (miR-181a, miR-30c, miR-30b) exhibited the highest values for more than one topological index (Supplementary Table 5A).

The genes targeted by miRNAs belonging to *cluster 1* were mostly controlled by 25 out of 38 miRNAs, and a few of these targeted the most genes (Supplementary Table 5B). By pathway enrichment analysis, the altered *TGF-beta* signalling pathway was regulated by TGFBR2 (miR-21, miR-214), SMAD3 (miR-21, miR-146a, miR-30b, miR-195, miR-503), and SMAD4 (miR-21, miR-146a, miR-181a, miR-181a^*^, miR-181b, miR-181c^*^, miR-181d) (Supplementary Table 5C).

#### PVAC

**Figure 4:**
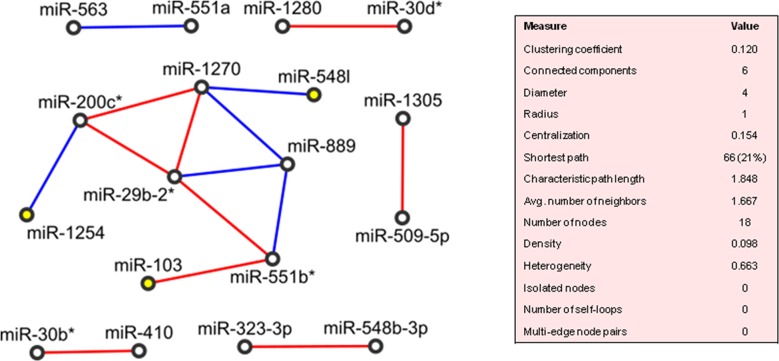
Correlation network between microRNA altered in tumour compared to normal tissue samples from patients with adenocarcinoma of papilla of Vater (PVAC) Direct and inverse correlations are in red and blue, respectively, and seeding nodes are colored in yellow.

The network wiring the 26 deregulated miRNAs of PVAC tumours was drawn in Figure 4. The clustering algorithm identified only one *cluster*, which included miR-1254, miR-200c^*^, miR-1270, miR-889, miR-551b^*^, miR-103, miR-29b-2^*^ and miR-548I (density: 0.533, p-value: 0.004) and so MiR-548I, miR-103 and miR-1254 worked as “seeding nodes” of the clustering method, and miR-29b-2-5p was directly correlated with two of the three seeding nodes (miR-103 and miR-1254). Additionally, 10 miRNAs were correlated in pairs: miR-551a/miR563, miR-410/miR30b^*^, miR-548b-3p/miR-323-3p, miR-1305/miR-509-5p, miR-30d^*^/miR-1280. The remaining 8 miRNAs did not show any significant correlation with any other miRNAs.

We found that 3 out of the 8 miRNAs (miR-103, miR-29b-2^*^, miR-200c^*^) controlled several genes (Supplementary Table 6A), which turned out to enrich different signalling pathways: *TGF beta* pathway (TGFBR2, APC, CDH1, CREBBP, EP300, SMAD3, TSC2), *altered TFG-beta SMAD dependent* signalling (TGFBR2, SMAD3, FBXW7), *p53* pathway and *transcriptional activation of cell cycle inhibitor p21* (TP53), and *E-cadherin* signalling events (CDH1), (Supplementary Table 6B).

### Networks matching

Four miRNAs were significantly deregulated both in PDAC and in PVAC. The regulation directions of miR-103 and miR-1254 were concordant, yet with different fold-changes values: miR-103 (PDAC: FC = 2.71; PVAC: FC = 15.43) and miR-1254 (PDAC: FC = -1.59; PVAC: FC =2.22). MiR-140-5p and miR-199b-5p were mildly altered in both the tumour entities, with opposite directions of regulation for the latter miRNA [miR-140-5p: FC=1.65 (PDAC), FC=1.43 (PVAC); miR-199b-5p: FC=1.57 (PDAC); FC=  -1.24 (PVAC)].

#### miR-103

**Figure 5:**
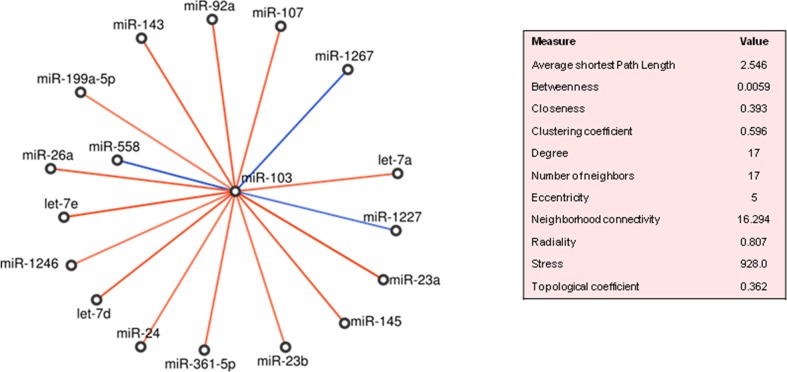
MiR-103 neighbourhoods in pancreatic ductal adenocarcinoma (PDAC) Direct and inverse correlations are in red and blue, respectively.

In PDAC, miR-103 was significantly correlated with 17 miRNAs that were not regulated less than 2 folds in tumour compared to normal tissues. Almost all correlations were positive, except from those involving miR-1227, miR-1267 and miR-558 (Figure 5). It is worth noticing that 15 on 17 miRNAs fell in the *cluster 2* (Supplementary Table 4). Topologically, miR-92a was in the top-ten ranked for *betweenness* (0.0499), while let-7a, let-7d and miR-145 were the most important in terms of *correlation coefficient* values (0.857, 0.727 and 0.705, respectively). MiR-103 targeted directly FBXW7, VCP, CHD1, PCDH17, HIP1, TGFBR2, TNF genes, while indirectly, through 11 of its 17 neighbour miRNAs, several more genes (Supplementary Table 7A). The majority of these genes were controlled by let-7d-5p, let-7a-5p, let-7e and miR-92a, and CDKN2A, FBXW7, TP53 and MYC were targeted by the most miRNAs. Some of the target genes, i.e., TGFBR2, SMAD3, SMAD4 regulated by (miR-103, miR-92a, miR-26a-5p, miR-145-5p, miR-143, let-7d-5p, miR-23a-3p, miR-107) turned out to participate to the *TGF-beta* signalling pathway in PDAC (Supplementary Table 7B).

In PVAC, miR-103 was positively correlated only with miR-551b^*^, which was in turn positively correlated with miR-29b-2^*^ and negatively with miR-889 (Figure 4). About predicted targets, the seven genes mentioned above were regulated by miR-103, whereas 23 genes were targeted by miR-29b-2^*^ (Supplementary Table 6A). These genes over-represented a number of signalling pathways: *TGF beta* pathway (TGFBR2, APC, CDH1, CREBBP, EP300, SMAD3, TSC2), *altered TFG-beta SMAD dependent* signalling (TGFBR2, SMAD3, FBXW7), *p53* pathway and *transcriptional activation of cell cycle inhibitor p21* (TP53), and *E-cadherin* signalling events (CDH1), (Supplementary Table 6B).

#### miR-1254

In PDAC, miR-1254 was directly correlated only with let-7g, which in turn was correlated with miR-221 and miR-92b. MiR-1254 did not target directly any known genes associated with the disease, and no target genes were found to be regulated by miR-92b. Conversely, let-7g was confirmed to target 9 critical genes (TP53, CDKN2A, FBXW7, FN1, MYC, TBX5, GLI1, TNF, ERCC4), and miR-221 resulted to control only 4 of these (TP53, CDKN2A, MYC, TNF).

In PVAC, miR-1254 was negatively correlated only with miR-200c^*^, which in turn was directly correlated with miR-29b-2^*^ and miR-1270 (Figure 5). As for miR-1254, no target genes of miR-1270 were reported to be directly associated to the disease. Conversely, miR-200c^*^ and 29b-2^*^ targeted several genes listed in Supplementary Table 5A.

Among the enriched signalling pathways, it emerges that the *DNA damage repair* pathway was shared by PDAC and PVAC. In details, the neighbourhood of miR-1254 was more involved in the regulation of this pathway: both miR-200c^*^ (correlated to miR-1254 in PVAC) and let-7g (correlated to miR-1254 in PDAC) targeted EP300 and ERCC4 genes, respectively.

#### miR-140-5p

In PDAC, miR-140-5p was correlated with 4 miRNAs (miR-939, miR-625^*^, miR-1249 and miR-27b-3p). Genes associated with the pancreatic cancer core signalling pathway were controlled only two of these miRNAs, namely miR-140-5p and miR-27b-3p. As shown in Supplementary Table 8A, these two miRNAs were predicted to regulate the same cancer-related target genes. By pathway enrichment analysis, several signalling pathways were uncovered: *p53* pathway, the *transcriptional activation of cell cycle inhibitor p21* (TP53), *E-cadherin* signalling events (CDH1), (Supplementary Table 8B).

Conversely, miR-140-5p did not correlate with any miRNA significantly deregulated in tumours, compared to normal tissues in PVAC patients. Therefore, the regulation of the above-mentioned signalling pathways might be imputed only to miR-140-5p in PVAC.

#### miR-199b-5p

In PDAC, miR-199b-5p was characterized by relevant topological values: average shortest path (3.8865) and topological coefficient (0.5238). Its expression was correlated with 3 miRNAs: directly with both miR-520a-3p and let-7d^*^, and inversely, with miR-196a. MiR-199b-5p did not directly target any known PDAC gene. However, part of its closest neighbourhood regulated several genes (Supplementary Table 9A). By pathway enrichment analysis, several signalling pathways were uncovered: *p53 pathway*, the *transcriptional activation of cell cycle inhibitor p21* (TP53), *E-cadherin* signalling events (CDH1), (Supplementary Table 9B).

Conversely, miR-199b-5p did not correlate with any miRNAs in the PVAC network.

### MiRNAs differentially expressed in PDAC *versus* PVAC

MiR-889 (FC=7.4) and miR-323-3p (FC=-2.12) were the most discriminating miRNAs between PDAC and PVAC. No genes associated with pathways involved in PDAC/PVAC were found to be targeted by miR-889, whereas for miR-323-3p two validated targets genes were identified (NTRK1 and TNF), which were involved in the biological process of *pain perception* (p= 1.421E-2).

The 27 miRNAs that resulted significantly altered between the matched-pairs of tissues of PDAC and PVAC significantly influenced the *Wnt* and *Hedgehog* signalling pathways, other than *MAPK*, *ErbB1* and *Notch* (already associated to PDAC) and *TFG-Beta* and *p53* (already associated to PVAC) signalling pathways.

## DISCUSSION

In the last years, several studies have investigated miRNA expression alterations in pancreatic cancer. However, the most were focused on PDAC while only a few investigated the expression profiles of miRNA in other pancreatic diseases, including PVAC [15–18].

Our study was designed to dig miRNA expression alterations out of PDAC or PVAC, and to figure out if these could be associated with distinctive signalling pathways in turn reflecting differences in pathogenesis and clinical behaviours of these two different entities of cancer.

Overall, PDAC and PVAC showed different miRNA alterations, with PDAC being more deregulated and probably a more complex disease. Changes in miRNA expression levels displayed a different direction in the two subgroups of patients. In PDAC samples, 68 out of the 101 altered miRNAs (67%) were over-expressed in tumours compared to normal tissues. These miRNAs may function as oncogenes promoting cancer development by negatively regulating tumour suppressor genes, whose aberrations were known to synergistically accelerate the progression of pancreatic carcinogenesis through pre-neoplastic lesions to adenocarcinoma [19, 20]. Conversely, more miRNAs were down-regulated in tumours compared to normal tissue samples from PVAC patients (19/26, 73% vs 7/26, 27%); the high percentage of under-expressed miRNAs in PVAC may function as tumour suppressor genes and may inhibit cancer by positively regulating oncogenes. With respect of the molecular changes, ampullary cancer was seen to be more similar to the intestinal cancer and characterized by alterations in oncogenes rather than in tumour suppressor genes [21, 22].

By comparing miRNA expression alterations in PDAC and PVAC we found that only 4 miRNAs (miR-140-5p, miR-103, miR-1254, miR-199b-5p) were deregulated in both the subgroups of patients. Conversely, a limited overlap between miRNAs significantly altered in both PDAC and PVAC emerged, suggesting the existence of tumour-specificity for miRNAs alteration in different entities of pancreatic cancer.

Furthermore, the expression of 27 miRNAs was significantly different in PDAC compared to PVAC. One of these, miR-323-3p, was shown to influence the biological process of pain perception. Pain has long been considered the most common symptom in patients with pancreatic disorders, and its presence in newly diagnosed patients with potentially operable pancreatic cancer is a predictor of resectability and survival [23]. However, the pattern of pain sensation process does not characterized different pancreatic tumours equally, while it was reported to depend on tumour type, anatomic localization and dignity of different pancreatic diseases [24]. This miRNA was already reported in literature for its association with painful events, such as ectopic pregnancy. Authors found that miR-323-3p was significantly increased in women experiencing abdominal pain/cramping and that received a diagnosis of ectopic pregnancy compared to those with intrauterine pregnancy and spontaneous abortion [25]. In our series miR-323-3p was altered in the opposite direction, so we can hypothesize that it can influence the pattern of pain sensation in PDAC and PVAC which clinically distinguishes these two tumour entities.

In order to identify the most relevant miRNAs in each tumour entity, and the signalling pathways in which there are involved, expression data were used to wire correlation networks both for PDAC and PVAC. MiRNA interactions in normal and tumour tissues were first taken into account in order to highlight the main alterations characterizing the two tissue types. Afterwards, with the intent to uncover those alterations that further exalt the differences between normal and tumour, the correlation networks were built by using miRNAs differentially expressed in matched pairs of tissues. By this approach, it was possible to identify three main subgroups of miRNAs either in PDAC and PVAC: miRNAs present only in the diseased network, those shared between normal and tumour network, and finally the most relevant miRNAs significantly altered in tumour compared to normal tissues.

In PDAC the diseased network was denser, more reachable and complex compared to the normal one. Indeed, it exhibited a shorter diameter, a higher centralization and a greater number of node neighbours, even though its local betweenness centrality was, in the average, lower than that of the nodes of the normal network. In general, the increased complexity of tumour compared to normal network might reflect the substantial reorganization of the controlling mechanisms fulfilled by these miRNAs in cancer. By comparing normal and tumour networks, we found that only a few miRNAs were not shared between the two networks, including 3 out of the 100 miRNAs connected in the tumour network (let-7d^*^, miR-1321, miR-373). By pathway enrichment analysis, ABC transporters signalling pathway emerged as regulated by miR-1321 and miR-373-3p.

Drug resistance is a major obstacle to the successful chemotherapy, and the ATP-binding cassette (ABC) transporter family members are the most common genes involved in the cancer multidrug resistance [26, 27]. We found that eight ABC proteins were targeted by the miR-1321 and miR-373-3p in PDAC network. Three out of them (ABCC2, ABCC5, ABCC8) have been already described for their association with drug resistance in pancreatic cancer [28–33]. Furthermore, a possible involvement of ABCC8 in pancreatic adenocarcinoma development and progression was recently reported [29].

By comparing normal and tumour networks in PDAC, we identified 97 miRNAs in common between the two networks including a core set of miRNAs that is partly or completely modulated by the disease (65 miRNAs which preserved unchanged correlations), and another set of 32 miRNAs which do not share any links between normal and tumour networks. In the latter subgroup we also identified 7 miRNAs characterized by high fold-change values. Among the most relevant pathways regulated by these subsets of miRNAs, we hypothesized that the Notch–mediated HES/HEY network, enriched only by the 7 miRNAs not sharing links, might be involved in the tumor progression compared to the pathways enriched by miRNAs belonging also to the subset of 65 miRNAs, which instead might be involved in the onset of the disease. In addition, according to the score values associated to each pathway, it was possible to suppose a sequential involvement for these signalling events in the carcinogenesis of pancreatic cancer: FOXO family and MAPK signalling pathways might intervene before, followed by regulation of nuclear SMAD2/3 signalling, while molecular events mediated by ERBB1 and c-MET might be deregulated later during the development of pancreatic cancer.

The importance of all these signalling pathways was supported by the literature. Most studies have revealed that the Notch activation has an oncogenic role for pancreatic cancer and is involved in cell proliferation, apoptosis, migration, invasion, metastases, and angiogenesis [34–41]. On the other hand, the mammalian forkhead members of the class O (FOXO) transcription factors are implicated as tumour suppressors in the regulation of several biological processes, including stress resistance, metabolism, cell cycle, apoptosis and DNA repair [42–48]. In relation to MAPK signalling, previous studies have shown that MAPK activity is required for PanIN formation and occurs early during the pancreatic transformation [49]. Similarly, SMADs have been identified as proteins that transduce the upstream signalling from TGF-Beta superfamily, thereby influencing cell proliferation, differentiation, and apoptosis [50, 51]. Finally, both the signalling pathways mediated ErbB1 and c-MET have been described for their associations with a more aggressive phenotype and poor prognosis in patients with pancreatic adenocarcinoma [52, 53].

Finally, the interactions network between miRNAs differentially expressed in tumour compared to normal tissues from PDAC corroborated the noteworthy complexity feature of the network and reflected the complexity of the disease at issue. Due to its size, this network has been clustered into five highly cohesive groups of miRNAs. The first cluster seemed to have a relevant role since a subset of miRNAs belonging to this cluster emerged as the top-five ranked for at least one topological index. Pathway analyses emphasized the importance of the signalling events mediated by TGF-beta in PDAC.

Disruption of normal TGF-beta pathway has been implicated in the pathogenesis and progression of pancreatic cancer, where it may play a dual effect as tumour suppressor and as a tumour promoter in normal and malignant cells, respectively [50]. In details, our finding showed that TGF-beta pathway was influenced by several miRNAs (miR-21, miR-214, miR146a, miR-30b, miR-195, miR503, miR181a-a^*^-b-c^*^-d) on 3 main genes (TFGBR2, SMAD3, SMAD4) in PDAC.

Networks analysis allowed to identify three main subgroups of miRNAs also in PVAC. Overall, the miRNAs and the signalling pathways closed to the tumour network did not correspond to those highlighted in the diseased network wired for PDAC in PVAC. Conversely, a partial overlap in relation to TGF-beta signalling events emerged from enrichment analysis based on either miRNAs shared between normal and tumour network and on those significantly altered in tumour compared to normal tissues samples in PVAC.

In details, the three miRNAs present only in the diseased network (miR-199b-5p, miR-323a -3p, miR-563) regulated a number of target genes involved in the LICAM1 and AXON guidance signalling pathways. Other authors reported that the increase of LICAM1 expression levels were associated with the chemo resistance and migratory phenotype of pancreatic cancer cells, and showed the significant correlation with the degree of perineural invasion of the tumour and the clinical course of patients [54, 55]. Similarly, the signals transmitted along the axons have been recently confirmed to be associated with diffusion and metastasis in pancreatic cancer [56, 57].

Among the miRNAs shared between the two networks, we found that the only common correlation was that between miR-1228^*^ and miR-1280; although Schopman et al. showed that the sequence annotated as miR-1280 is likely to be a fragment of a tRNA [58], these two miRNAS were likely to initiate and drive the correlation chains for normal and tumour tissues, respectively. In relation to miR-889, even if this miRNA did not target any genes associated with pancreatic cancer, its changes in either direction and neighbouring in the two networks may be the sign of an important change in the functional targeting between healthy and tumour tissues. Conversely, miR-103 was the only miRNA among those with a high fold-change value that targeted genes associated with pancreatic cancer; it was in a prominent topological position in normal network, whereas it was the only miRNA to be highly expressed and is likely to drive the longest chain of consecutive inverse correlations in the tumour network. We found that miR-103 influenced, *TFG-beta* pathways (TGFBR2, APC, CDH1, CREBBP, EP300, SMAD3, TSC2), *altered TGF-beta SMAD dependent* signalling (TGFBR2, SMAD3, FBXW7), and *E-cadherin* signalling events (CDH1) in PVAC.

Noteworthy, miR-103 seemed to have a peculiar role even in PDAC, where it was an important hub of the interactions network between miRNAs differentially expressed in tumours compared to normal tissues; therefore, even if it did not directly modulate many genes, it might exert a relevant influence to the target genes of its neighbour miRNAs, most of which belong to the cluster 2 of the network. The shared centrality of miR-103 in the two cancer entities was accompanied by an overlap in the signalling pathways associated with this miRNA. As uncovered by enrichment analysis, miR-103 influenced the signalling pathway mediated by TFG-Beta in PDAC and PVAC. However, while in PDAC more miRNAs connected to miR-103 regulated specific target genes, in PVAC miR103 exerted its direct influence on a larger number of genes.

In relation to these genes, recent studies have revealed a molecular association between TGF-beta/SMADs signalling pathways and tuberin (TSC2), a tumour suppressor gene involved in cell growth and differentiation, and in cell cycle progression [59]. SMADs were also reported to interact with CBP and p300, proteins having histone acetyl transferase and histone deacetylase activities, and to play an important role in cell proliferation and differentiation [60]. The association of SMAD2 and SMAD3 with APC gene was reported to contribute as antagonist of the events mediated by TGF-beta [61]. Within the SMAD signal transduction pathway, FBXW7 gene enhances TGFβ-dependent transcription by inactivating the repressor transcriptional repressor TGIF1 [62–64]. Furthermore, CDH1, the adhesion molecule involved in cell-to-cell cohesion, cell-to-cell recognition, and epithelial polarity, is known to be regulated by TGFβ in pancreatic ductal adenocarcinoma [65]. It is involved in tumour progression and in prognosis of patients with pancreatic cancer [66–71].

Evaluation of interactions network between miRNAs differentially expressed in tumour compared to normal pancreatic tissues from PVAC highlighted that other two miRNAs (miR-200c^*^ and miR-29b-2^*^) contributed with miR-103 to enriching TFG-beta and altered TGF-beta SMAD dependent, signalling events E-cadherin, pathway and signals mediated by p53 and p21. Once again, data showed an overlap in the regulation of TGF beta pathway. However, it must be stressed that the signalling events at issue were regulated by different miRNAs and in turn by different target genes in PVAC compare to PDAC.

Noteworthy, another peculiarity in PVAC concerned the topological organization of miRNAs forming two closed groups in the normal network. Since these closed groups did not persist in the diseased network, they might be the sign of an important change in functional targeting between healthy and tumour tissues. Furthermore, it was hypothesized that these closed groups can identify “functional collaborators”: genes and biological processes regulated by miRNAs can be associated with each other; alternatively it was possible to speculate that the effects of a miRNA on target genes and their respective signalling pathways occur according to miRNA expression, which in turn may depend on the correlation between the expression levels of miRNAs in the closed groups [72–74]. In details, miR-548b-3p of the first closed group regulated DICER1, involved in the biogenesis of miRNAs themselves;Recently, a potential oncogenic role for DICER1 in pancreatic cancer initiation was also reported [75–77]. On the other hand several genes were influenced by miR-140-5p of the second closed group. These genes significantly enriched the signalling events mediated by p53, p21 and E-cadherin. The importance of these pathways in pancreatic carcinogenesis are well documented [68, 69, 72, 78, 79].

In relation to the pathways influenced by miR-140-5p, a difference between PDAC and PVAC emerged:miR-140-5p acts independently and directly in PVAC, whereas its neighbour miR-27b-3p regulates of the same signalling events in PDAC.

In addition, we found that the signalling pathways mediated by miR-140-5p were influenced also by miR-199b-5p in PDAC: its three neighbours (miR-196a, let7d^*^, miR-520a-3p) regulated several target genes (TP53, CDH1, TSC2, CREBBP, TGFBR2, SMAD3, SMAD4) involved in these signalling events.

On the other hand, miR-1254 exemplified the case of a miRNA involved in the same signalling pathways in the two entities of pancreatic cancer, albeit thought different neighbours influencing different target genes. Indeed, either ERCC4 and EP300, targeted by let-7g and miR-200c^*^ in PDAC in PVAC respectively, influenced the DNA damage repair pathway. Evidences have revealed that ERCC4 expression levels are correlated with cancer risk, progression, response to chemotherapy, and clinical outcome of different tumours, such as head and neck cancer, suggesting that altered ERCC4 expression may lead to altered DNA repair capacity, thereby modulating cancer susceptibility [80]. Similarly, EP300 is a transcriptional co-activator that mediates many transcriptional events including DNA repair [81].

Finally, the enrichment analysis was performed by using the 27 miRNAs significantly altered in matched pairs of tissues from PDAC compared to those from PVAC, in order to predict a role for miRNAs in distinguishing between PDAC and PVAC. A part from conforming the involvement of miRNAs in some of the pathways previously associated with PDAC or PVAC, our findings uncovered the regulation of the Wnt and the Hedgehog signalling pathways. Wnt signalling events were reported in the literature for their association with the carcinogenesis and progression of pancreatic cancer [82]. Similarly, activation of Hedgehog signalling pathway influences cell proliferation and cell cycle and thus has a role for initiation of pancreatic cancer either alone or in a K-Ras dependent way [83, 84].

**Figure 6:**
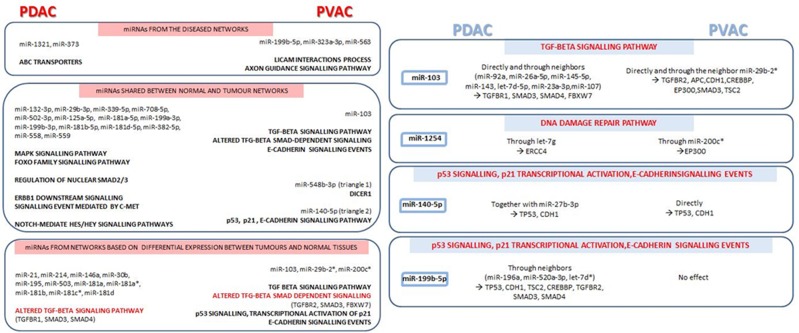
Main signalling pathways, together with miRNAs and respective target genes, emerged from microRNA co-expression networks in pancreatic ductal adenocarcinoma (PDAC) compared to Vater's papilla adenocarcinoma (PVAC) Shared signalling events, regulated by different miRNAs and/or genes in PDAC and PVAC, are highlighted in red.

In general, PDAC was characterized by a more consistent miRNA alteration compared to PVAC, and a more complex interactions networks were found either for miRNAs connected in tumour tissues and for those altered in tumours compared to normal pancreatic samples. However, with few exceptions especially in relation to the signalling events regulated by miRNAs connected only in the tumour networks and for those emerged from miRNAs shared between tumour and normal networks, our findings show a good overlap between the signalling pathways influenced by miRNAs in the two tumour types. Nevertheless, the shared signalling pathways were regulated by different miRNAs and/or genes in PDAC and PVAC (Figure 6).

Overall our findings reflect the specificity of miRNAs expression patter within different entities of pancreatic cancer, and suggest a role for these molecules in the regulation of a limited core signalling pathways in the biology landscape of PDAC and PVAC.

Father studies are needed to corroborate our data from co-expression networks. In details, the biological effect of specific miRNAs should be functionally validated *in vitro* to verify the interaction between miRNA and mRNA target, their co-expression, and their effect on the predicted protein expression up to the alteration of respective signalling pathways.

## MATERIALS AND METHODS

### MiRNA profiling datasets

The main cohort of this study is made of two groups of patients, 9 PDAC and 4 PVAC, enclosed in our previous miRNAs expression profiling study on matched-pairs of normal and tumour tissues samples from 17 patients with different histological types of pancreatobiliary cancer (including, apart from PDAC and PVAC, adenocarcinoma arising from the biliary epithelium) [85].

Tissue samples were obtained from patients who underwent pancreatic resection for PDAC or PVAC, and before any chemotherapy had been initiated. In details, specimens were harvested during surgery after extemporaneous anatomopathological test for evaluation of both the tumour and the adjacent non-affected pancreatic tissue which were then taken and stored separately. In addition, later pathological evaluations ascertained the origin from duodenal mucosa for all the PVAC samples.

Gene expression was profiled with Affymetrix GeneChip miRNA 2.0 Arrays and made available from ArrayExpress (E-MTAB-753). Symbols of miRNAs were converted to their most recent forms and annotated with their MIMAT IDs using the aliases tracking system provided by miRBase (Supplementary Information #Sheet B).

An external dataset (E-GEOD-60978), made of 52 PDAC and 6 normal tissues profiled by Agilent 031181 Unrestricted Human miRNA V16.0 Microarray 030840 platform, was used to crosscheck the evidences of expression correlation found in this work. No public datasets of miRNA expression profiles of PVAC were found, therefore a similar analysis for our PVAC counterpart could not be made.

### Statistical analysis

In order to identify differentially expressed miRNAs between paired normal and PDAC/PVAC tumour tissues, paired *t*-tests were performed controlling for false discovery rate (fdr) allowing us to rank miRNAs according to their p-values. In each cohort, correlations between miRNAs expression were estimated using Spearman coefficient, in order to account for non-normal data distribution. These correlation matrices were then used for bioinformatics analyses. A p-value of 0.05 was considered for statistical significance. All analyses were performed using SAS Release 9.1 (SAS Institute, Cary, NC, USA).

### Geometric and topological analysis

Similar to the analysis workflow followed in [85] and [86], we built undirected and weighted graphs of miRNA-to-miRNA based on correlation data. Nodes and edges of graphs represented miRNAs and their correlation values, if significant, respectively. Edges were not oriented since correlation is a symmetric measure, and were weighted with the Spearman coefficient. Initially, graphs were drawn separately for normal and tumour tissues. Then, only differentially expressed miRNAs between both tissues for both tumour entities were considered. Networks were drawn and analysed by Cytoscape 3.1.0 [87] and by a custom standalone tool written in C# and built over the library NodeXL 1.0.1.317.

#### Global geometries

Graphs connectivity, reachability and cohesiveness were measured by well-known global topological indices. We first counted the number of *connected components*, which is the number of groups of nodes that are pairwise connected. A lower number of connected components suggest a stronger connectivity. Then, we measured the *diameter*, meant as the longest among all the shortest paths of a network, and the *characteristic path length*, also known as the *average shortest path length*, which gives the expected distance between two connected nodes. We further considered the *network centralization*, which is 1 if a network resembles a star or 0 if it is decentralized, the *average number of neighbours*, which indicates the average connectivity of a node, and its normalized version: the *network density*. The density is a value between 0 and 1. It shows how densely a network is populated with edges. A network that contains no edges and solely isolated nodes has a density of 0. In contrast, the density of a clique is 1 [88, 89]. Finally, we considered the *network heterogeneity* that reflects the tendency of a network to contain central and highly connected nodes, also known as *hubs*.

#### Local topological metrics

Critical miRNAs were recognized because of their topological measures: *degree*, *betweenness*, *closeness* and *clustering coefficient* [90–92]. All of them are based on the enumeration of links or shortest paths, whose length is calculated by summing the inverse weights of the traversing edges. The idea is that highly correlated miRNAs minimize the distance between nodes. Thus, while *degree* centrality relies on the fact that important nodes are those with the largest number of ties to other nodes, the *betweenness* index measures the influence a node has over the indirect correlation between even distant not-neighbour nodes. *Closeness* highlights nodes that are particularly ‘close’ to other nodes, or ‘reachable’ from other nodes. The shorter the geodesic distance from a node to other nodes, the higher its *closeness* centrality. On the contrary, the *clustering coefficient* measures the degree to which miRNAs tend to cluster together. Scatter plots summarizing the distributions of local topological indices are available in Supplementary Figure 1.

#### Cluster-based functional enrichment analysis

Important groups of miRNAs were identified by the Molecular Complex Detection (MCODE) clustering algorithm [93]. Weighting each miRNA by the local neighbourhood density and outward traversal from locally dense seed miRNAs, we isolated several dense regions that shared molecular targets and putatively cooperated to the fulfilment of a common biological process.

MirTarBase [94] and MirWalk [95] were queried (April 2015) to get experimentally validated target genes of selected miRNAs. Clashes of names and aliases were resolved by querying miRBase [96]. Target genes underwent functional enrichment analysis against the Gene Ontology FAT sub-set. Results obtained with DAVID [97] and ToppGene [98] web services were crosschecked with Babelomics [99] and considered if Bonferroni-corrected significance levels did not exceed 5%.

## SUPPLEMENTARY MATERIALS FIGURE AND TABLES






