# Supplementary material for: MicroRNA co-expression networks exhibit increased complexity in pancreatic ductal compared to Vater's papilla adenocarcinoma
Source: Oncotarget. 2017 Oct 31;8(62):105320–39. doi: 10.18632/oncotarget.22184 (PMC5739641; doi:10.18632/oncotarget.22184)
Supplement: Supplementary file 1 [file oncotarget-08-105320-s001.pdf]

MicroRNA co-expression networks exhibit increased complexity in pancreatic ductal compared to Vater's papilla adenocarcinoma

SUPPLEMENTARY MATERIALS

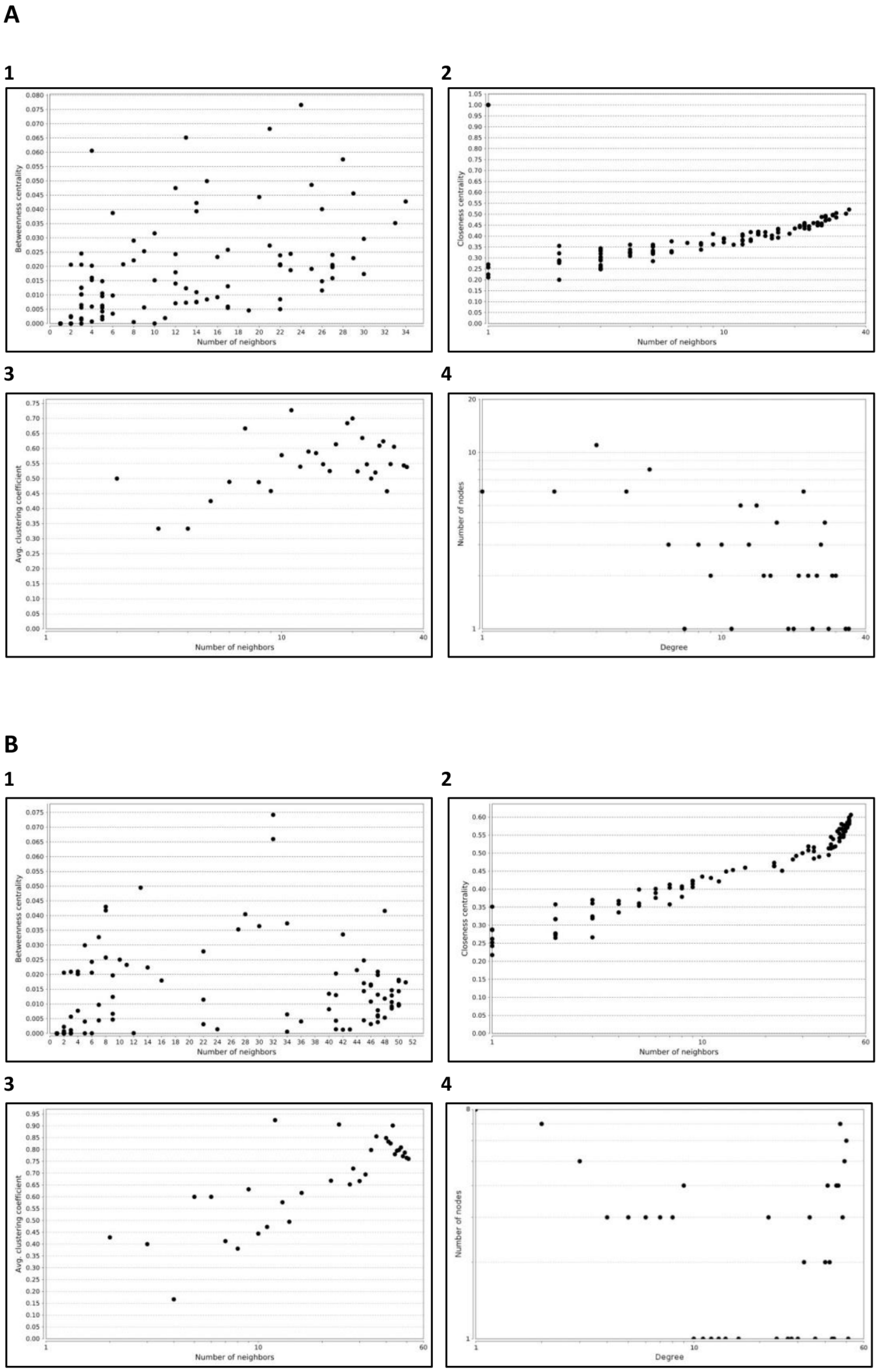

Supplementary Figure 1: Graphical representation of betweenness (1), closeness (2), clustering coefficient (3), as a function of each node neighbour, and degree (4), as a function of each node for: **(A)** PDAC network of correlated and differentially expressed genes; **(B)** PDAC correlation network (normal matched samples);

C

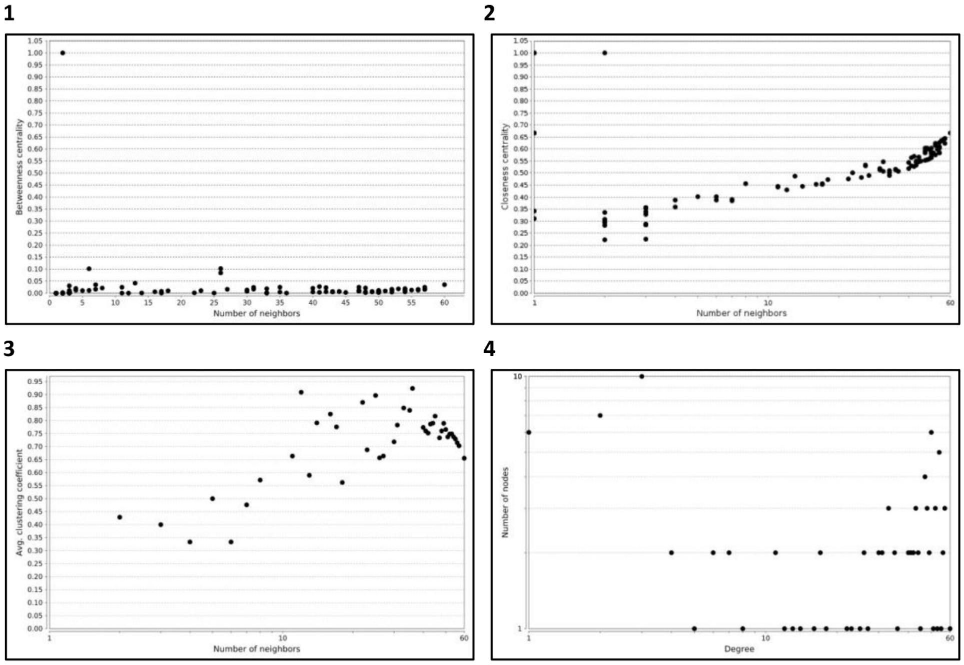

D

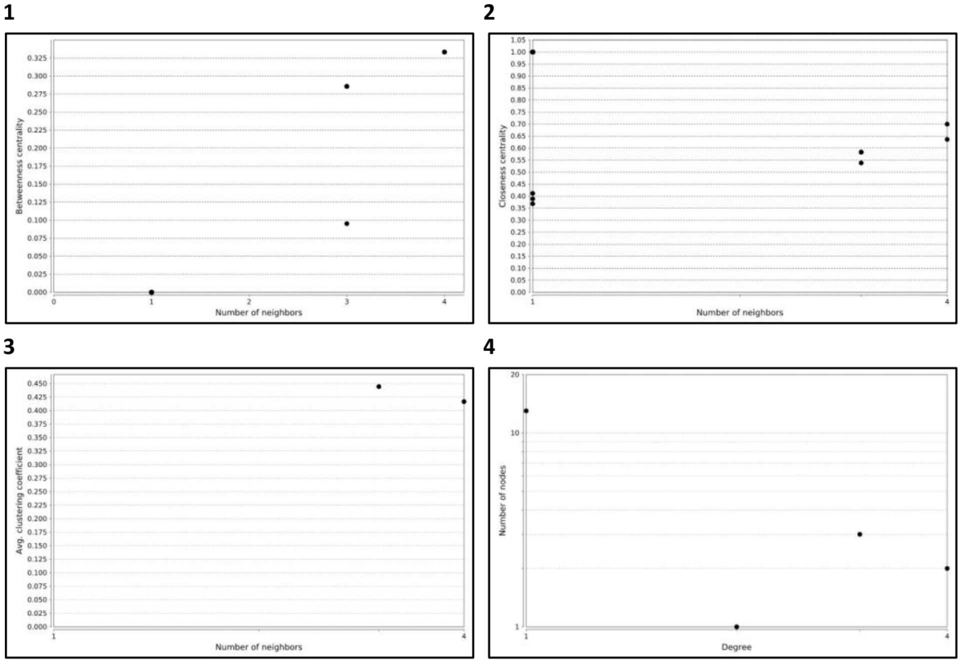

Supplementary Figure 1: (*Continued*) Graphical representation of betweenness (1), closeness (2), clustering coefficient (3), as a function of each node neighbour, and degree (4), as a function of each node for: **(C)** PDAC correlation network (tumor only); **(D)** PVAC network of correlated and differentially expressed genes.

E

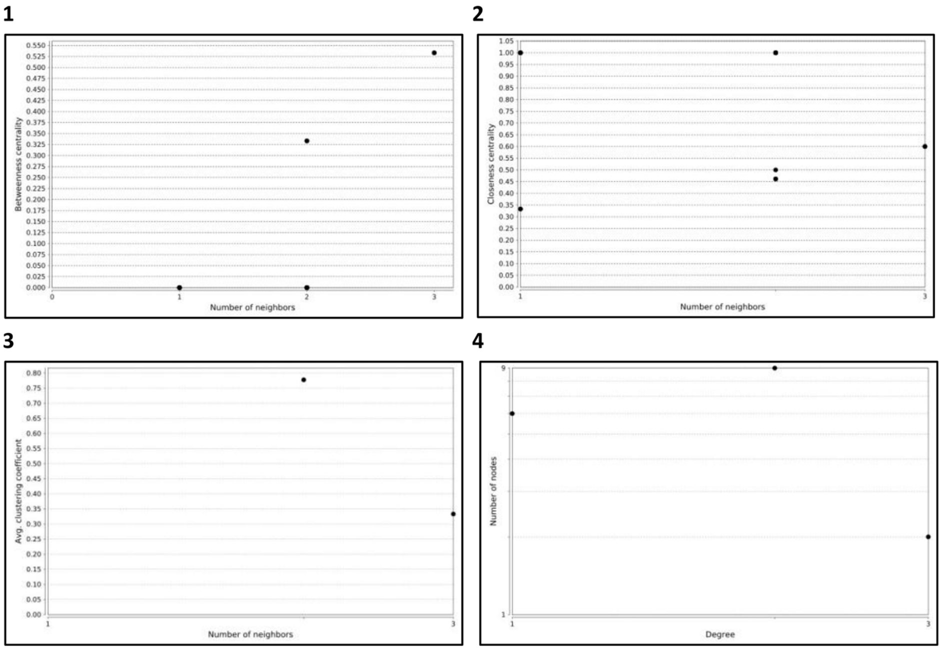

F

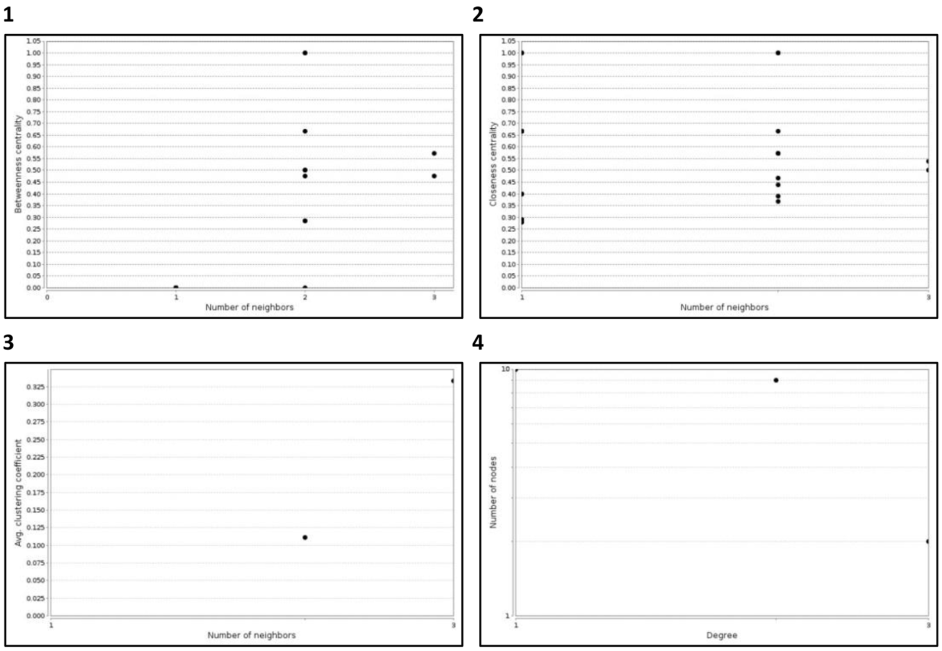

Supplementary Figure 1: ***(Continued)*** Graphical representation of betweenness (1), closeness (2), clustering coefficient (3), as a function of each node neighbour, and degree (4), as a function of each node for: **(E)** PDAC correlation network (normal matched samples); **(F)** PVAC correlation network (tumor only).

Supplementary Table 1: Alteration of human microRNA in other organisms.

See Supplementary File 1

Supplementary Table 2: MiRNAs shared between tumour and normal networks in pancreatic ductal adenocarcinoma (PDAC).

See Supplementary File 1

Supplementary Table 3: Signalling pathways enriched by miRNAs specific for tumour network in pancreatic ductal adenocarcinoma (PDAC).

See Supplementary File 1

Supplementary Table 4: MiRNAs clusters in pancreatic ductal adenocarcinoma (PDAC).

See Supplementary File 1

Supplementary Table 5: MiRNAs cluster 1 in pancreatic ductal adenocarcinoma (PDAC).

See Supplementary File 1

Supplementary Table 6: Target genes and signalling pathways regulated by miR-103, miR-29b-2*, miR-200c* in adenocarcinoma of papilla of Vater (PVAC).

See Supplementary File 1

Supplementary Table 7: Target genes and signalling pathways regulated by miR-103 and its neighbourhoods belonging to miRNAs *cluster 2* in pancreatic ductal adenocarcinoma (PDAC).

See Supplementary File 1

Supplementary Table 8: Target genes and signalling pathways regulated by miR-140-5p and its neighbourhood miR-27b-3p.

See Supplementary File 1

Supplementary Table 9: Target genes and signalling pathways regulated by neighbourhoods of miR-199b-5p in pancreatic ductal adenocarcinoma (PDAC).

See Supplementary File 1

Supplementary Information:

See Supplementary File 2
